# Adenosquamous carcinoma of the ampulla of Vater: a case report and literature review

**DOI:** 10.1186/s12957-015-0709-0

**Published:** 2015-09-29

**Authors:** Sojun Hoshimoto, Koichi Aiura, Masaya Shito, Toshihiro Kakefuda, Hitoshi Sugiura

**Affiliations:** Department of Surgery, Kawasaki Municipal Hospital, Kawasaki, 210-0013 Kanagawa Japan; Department of Pathology, Kawasaki Municipal Hospital, Kawasaki, 210-0013 Kanagawa Japan

**Keywords:** Adenosquamous carcinoma, Carcinoma of the ampulla of Vater, Pancreaticoduodenectomy

## Abstract

**Background:**

Adenosquamous carcinoma of the ampulla of Vater is extremely rare, and its clinicopathological features are limited and described in few previous case reports. Here, we report curative resection of adenosquamous carcinoma of the ampulla of Vater at an early stage.

**Case presentation:**

An 81-year-old woman was referred to our hospital for investigation of the frequent elevation of hepatic and biliary enzymes and dilatation of the intrahepatic bile ducts. Preoperative examinations revealed an exposed reddish tumor in the ampulla of Vater, which was diagnosed on biopsy to be adenocarcinoma with squamous cell carcinoma component. Pylorus-preserving pancreaticoduodenectomy with regional lymph node dissection was performed. Pathological examinations revealed the presence of two malignant components in the lesion, including poorly differentiated tubular adenocarcinoma and squamous cell carcinoma, without invasion beyond the sphincter of Oddi or into the duodenal submucosa. These squamous cell carcinoma and adenocarcinoma components in the tumor comprised approximately 30 and 70 % of the lesion, respectively. No metastasis into regional lymph nodes was observed, and the patient experienced no tumor recurrence or metastasis until 20 months after surgery.

**Conclusion:**

We identified only six reported cases of adenosquamous carcinoma of the ampulla of Vater in the English literature, and all of these patients died of recurrence within 14 months after surgery. To the best of our knowledge, this is the first report of adenosquamous carcinoma of the ampulla of Vater that was curatively resected at an early stage. Although more number of studies on clinicopathological findings are required to determine the appropriate surgical indication, we suggest that surgery remains the mainstay therapy for adenosquamous carcinoma of the ampulla of Vater detected at an early stage.

## Background

Adenosquamous carcinoma (ASC) is characterized by variable combinations of two malignant components, including adenocarcinoma and squamous cell carcinoma (SCC). Adenocarcinomas account for most malignancies of the ampulla of Vater; however, ASC is a rare condition. Although the proportion of the aforementioned two components varies, adenocarcinoma usually predominates. According to the WHO classification of tumors of the digestive system, ASC is shown to comprise at least 25 % of the SCC component in the tumor [1].

ASCs in other primary sites that normally have glandular epithelium, such as the pancreas, stomach, colon and rectum, breast, and prostate, are also rare and are considered to be clinically more aggressive, with worse prognosis than their adenocarcinoma counterparts [2–8]. Most studies on ASC in the ampulla of Vater have been small series or single case reports because of its rarity. Therefore, clinicopathological features and outcomes of this entity remain unclear and no therapeutic strategies have been established. Here, we report a case of ASC of the ampulla of Vater that was curatively resected at an early stage, and review the reported cases of this unusual entity.

## Case presentation

**Fig. 1:**
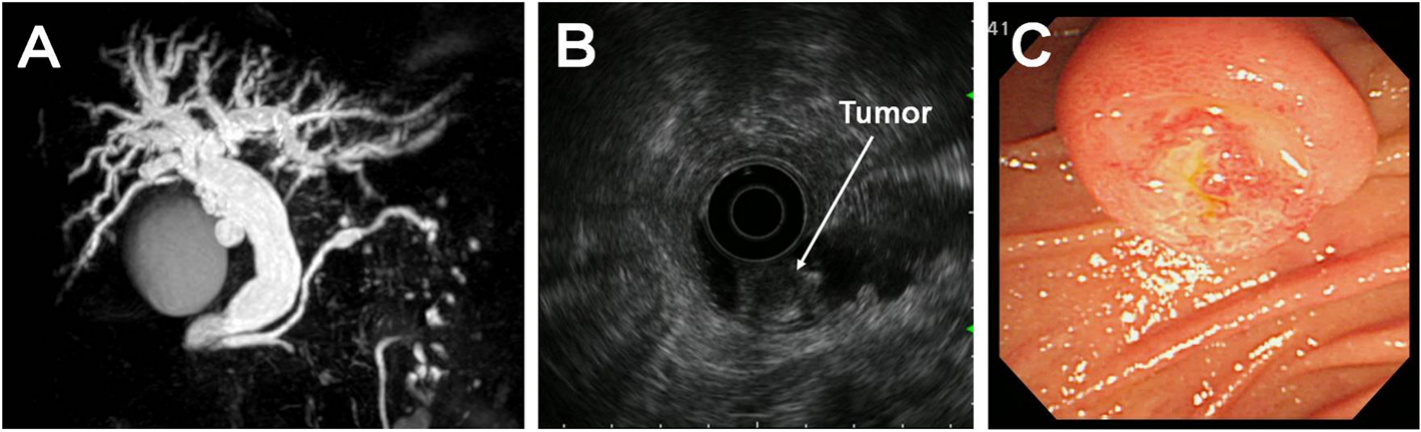
**a** Magnetic resonance cholangiopancreatography showed dilatation of the intra- and extrahepatic bile ducts and the main pancreatic duct without any stricture. **b** Endoscopic ultrasonography showed a hypoechoic mass in the ampulla of Vater located on the inside of the duodenal wall without invasion of the duodenal muscularis propria. **c** Duodenoscopy revealed an exposed reddish tumor at the ampulla of Vater

**Fig. 2:**
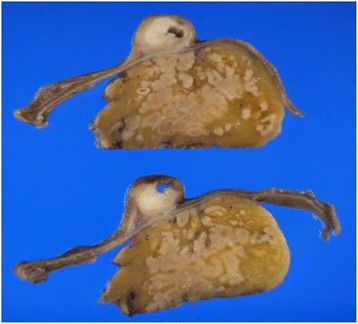
Macroscopic findings. A whitish and solid exposed-type tumor, 11 × 8 mm in size, was observed in the ampulla of Vater

**Fig. 3:**
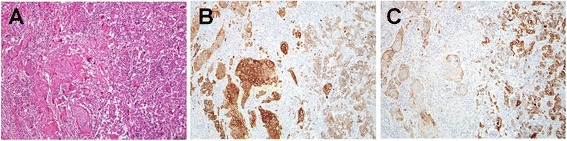
Histopathological and immunohistochemical findings. **a** Hematoxylin and eosin staining. **b** CK5/6 was strongly expressed in the squamous cell carcinoma component. **c** CK7 was expressed in the adenocarcinoma component (original magnification ×15)

An 81-year-old Japanese woman with a medical history of type II diabetes mellitus and arteriosclerosis obliterans was referred to our hospital for investigation of the frequent elevation of hepatic and biliary enzymes and dilatation of the intrahepatic bile ducts, which were identified by her family doctor during abdominal ultrasonography. The patient did not complain of abdominal pain or decreased appetite. Laboratory examinations revealed slightly elevated biliary enzymes, including 413 IU/L alkaline phosphatase (normal, 104–338 IU/L) and 184 IU/L $\gamma$-glutamyl transpeptidase (normal, 9–28 IU/L); however, other serum chemistry data were within normal limits. Levels of the serum tumor markers carcinoembryonic antigen and carbohydrate antigen 19-9 were also within normal limits. Contrast-enhanced abdominal computed tomography and magnetic resonance cholangiopancreatography showed dilatation of the intra- and extrahepatic bile ducts and the main pancreatic duct without any strictures (Fig. 1a), suggesting the presence of an ampullary tumor. Neither lymph node enlargement nor metastasis was observed. Endoscopic ultrasonography showed a hypoechoic mass of 9 × 9 mm in the ampulla of Vater (Fig. 1b). The tumor was located on the inside of the duodenal wall and had not invaded the duodenal muscularis propria, pancreas, common bile duct terminal, or main pancreatic duct. Duodenoscopy revealed a bulging oral protrusion because of the dilated distal bile duct and an exposed reddish tumor at the ampulla of Vater (Fig. 1c), which was diagnosed on biopsy to be adenocarcinoma with the SCC component. Endoscopic retrograde cholangiopancreatography (ERCP) showed dilatation of the upstream bile duct and main pancreatic duct, and ERCP followed by transpapillary biliary intraductal ultrasonography revealed a hypoechoic tumor confined within the ampullary portion of the duodenum, the common channel of the ampulla, and the ampullary portion of the bile duct area. However, no extension into the ampullary portion of the pancreatic duct or the distal bile duct was observed. Because the patient showed the onset of acute cholangitis secondary to ERCP, endoscopic retrograde biliary drainage was performed using a plastic stent on the day after ERCP. Based on these findings, the lesion was diagnosed as early-stage carcinoma of the ampulla of Vater with the SCC component without extension along the distal bile duct or main pancreatic duct. Subsequently, pylorus-preserving pancreaticoduodenectomy (PPPD) with regional lymph node dissection was performed. Macroscopic examinations revealed a whitish and solid exposed-type tumor, 11 × 8 mm in size in the ampulla of Vater (Fig. 2). Pathological examinations showed the presence of two malignant components, including poorly differentiated tubular adenocarcinoma and SCC without invasion beyond the sphincter of Oddi or into the duodenal submucosa (Fig. 3a). SCC and adenocarcinoma components in the tumor comprised approximately 30 and 70 % of the tumor mass, respectively. No regional lymph node metastases or lymphovascular or perineural infiltrations were observed. Immunohistochemistry (IHC) analyses of the squamous marker cytokeratin (CK)-5/6 showed strong positive expression in the SCC component and slight expression in the adenocarcinoma component (Fig. 3b). In contrast, the adenocarcinoma marker CK-7 was strongly detected in the adenocarcinoma component and weakly detected in the SCC component (Fig. 3c). The postoperative course was uneventful and the patient experienced no tumor recurrence or metastasis until 20 months following surgery.

## Discussion

Although the histogenesis of ASC remains unclear, the four prevailing hypotheses describe (1) pluripotent stem cells capable of inducing the malignant transformation of both cell types, (2) squamous metaplasia in the intestinal mucosa, (3) malignant squamous metaplastic transformation of the adenocarcinoma, and (4) collision of the two malignant tumors [9]. It is currently accepted that adenosquamous carcinomas may occur through metaplastic malignant squamous transformation of adenocarcinomas [10]. Accordingly, in the present pathological examination, the SCC component was heterogeneously distributed and IHC staining for squamous cell carcinoma and adenocarcinoma markers clearly separated discrete SCC and adenocarcinoma components, with no bidirectional differentiation of individual tumor cells. Moreover, neither squamous metaplasia in the uninvolved adjacent epithelium nor a transition from squamous metaplasia to squamous cell carcinoma could be identified. These observations are consistent with the theory of metaplastic malignant squamous transformation of an adenocarcinoma.

PubMed was searched using the terms “ampulla,” “papilla,” or “Vater,” and “adenosquamous carcinoma” and six reported cases of ASC of the ampulla of Vater were retrieved from the English literatures [9, 11, 12]. Demographic and clinical characteristics of all studied patients, including the present patient, are summarized in Table 1. The average age at diagnosis was 62.0 ± 17.4 years (range, 47–82), and five of seven patients (71 %) were men. In the present case, carcinoma of the ampulla of Vater was diagnosed during the work-up for elevations of serum hepatobiliary enzymes. All other patients presented with symptoms such as abdominal pain and jaundice, which were similar to the symptoms of conventional adenocarcinomas of the ampulla of Vater. Preoperative endoscopic biopsy identified the SCC components in four cases (57 %), whereas the diagnosis of ASC was made after surgical resection in three cases (43 %). Surgical procedures performed were pancreaticoduodenectomy, including PPPD, in six cases (86 %) and ampullectomy in one case (14 %). The mean tumor size in the four cases with relevant data was 26.8 ± 12.9 mm (range, 11–40), and lymph node metastases were identified in three of six cases with relevant data (50 %). Overall survival in all seven cases ranged from 6 to 20 months, and six of seven patients survived within 14 months after surgical resection. In contrast, the present case survived for 20 months without any evidence of recurrence.

**Table 1:** Reported cases of adenosquamous carcinoma of the ampulla of Vater in the English literature

Year	Author	Age	Gender	Symptom	Size (mm)	Biopsy	Treatment	LN mets	UICC stage	Proportion of SCC	Prognosis (months)	
2002	Ueno	47	M	Jaundice, general fatigue	22	SCC	PD	(+)	III	75 %	10	Dead
2013	Yang	64	M	Jaundice, abdominal pain	34	ADC	PD	(+)	IIB	SCC>>ADC	6	Dead
2013	Yang	82	M	Jaundice	NM	SCC (−)	Ampullectomy	(−)	IB	NM	14	Dead
2013	Yang	68	M	Jaundice, abdominal pain	NM	SCC (−)	PD	(+)	III	NM	7	Dead
2013	Yang	34	F	Jaundice, abdominal pain	NM	SCC (−)	PD	(−)	III	NM	10	Dead
2014	Kshirsagar	58	M	Jaundice, abdominal pain	40	SCC	PD	NM	NM	NM		NM
	Present case	81	F	No symptoms	11	ADC + SCC	PPPD	(−)	IA	30 %	20	Alive

*SCC* squamous cell carcinoma, *ADC* adenocarcinoma, *PD* pancreaticoduodenectomy, *PPPD* pylorus-preserving pancreaticoduodenectomy, *NM* not mentioned

ASC is regarded as a more clinically aggressive tumor with less favorable prognosis than adenocarcinomas of other origins [2–8]. Imaoka et al. [13] reported that the median overall survival of patients with ASC of the pancreas was nearly half that of patients with pancreatic ductal adenocarcinomas. Prognosis of the reported cases of ASC of the ampulla of Vater except the present case was poor; however, because of the limited number of cases, more studies on clinicopathological findings are necessary to analyze whether the prognosis of ASC was less favorable than that of the conventional adenocarcinomas of the ampulla of Vater as well as other origins.

Few reports describe associations between the proportion of SCC components and tumor progression [14]. However, SCC components grow more aggressively than adenocarcinoma components in animal models [15]. Moreover, several studies report significantly shorter doubling times of SCCs than adenocarcinomas [16, 17], suggesting that the proportion of SCC components in ASC increases with tumor progression and may be associated with prognosis. Accordingly, two reported cases of ASC of the ampulla of Vater with predominant SCC component died of recurrence within 10 months after surgery [9, 12]. However, the Ki-67 index of poorly differentiated SCC and adenocarcinoma components in the present case were 33 and 34 %, respectively, indicating a similar proliferative potential of SCC and adenocarcinoma components. Yang et al. [9] demonstrated no survival benefits of surgical interventions and promoted chemoradiation as the first choice of treatment for ASC of the ampulla of Vater if the diagnosis of ASC could be made before surgery. However, pathological observations of the present case showed limited tumor invasion within the sphincter of Oddi and no evidence of regional lymph node metastasis or perineural or angiolymphatic invasions. Moreover, the SCC component comprised only 30 % of the tumor, presumably resulting in continued survival until 20 months after surgery without evidence of recurrence. Therefore, we suggest that surgery remains the mainstay therapy for ASC of the ampulla of Vater, although diagnosis of the condition during the early stages when the adenocarcinoma component predominates over the SCC component is critical.

## Conclusions

Here, we report an unusual case of ASC of the ampulla of Vater. The subsequent literature review revealed that all the reported cases of ASC of the ampulla of Vater carried poor prognosis. To the best of our knowledge, this is the first report of ASC of the ampulla of Vater that was curatively resected at an early stage. Because of its rarity, more number of studies on clinicopathological findings are required to determine the association between the proportion of the tumor comprising SCC and progression, clinical outcome, and appropriate surgical indication. Nonetheless, the present case supports early detection and the use of surgery as the only curative treatment option for this rare entity.

## Consent

Written informed consent was obtained from the patient to allow publication of this case report and any accompanying images. A copy of the written consent is available for review by the Editor-in-Chief of this journal.

## Abbreviations

ASC: Adenosquamous carcinoma
CK: Cytokeratin
ERCP: Endoscopic retrograde cholangiopancreatography
IHC: Immunohistochemistry
PPPD: Pylorus-preserving pancreaticoduodenectomy
SCC: Squamous cell carcinoma
